# Severity of illness and the weekend mortality effect: a retrospective cohort study

**DOI:** 10.1186/s12913-020-5029-6

**Published:** 2020-03-04

**Authors:** Eric Bressman, John C. Rowland, Vinh-tung Nguyen, Beth G. Raucher

**Affiliations:** 1 Department of Medicine at Mount Sinai, 1 Gustave L Levy Pl, New York, NY 10128 USA; 2 Department of Population Health Science and Policy at Mount Sinai, 1425 Madison Ave, New York, NY 10128 USA

**Keywords:** Weekend mortality, Weekend admission, Severity of illness

## Abstract

**Background:**

Weekend admission to the hospital has been found to be associated with higher in-hospital mortality rates, but the cause for this phenomenon remains controversial. US based studies have been limited in their characterization of the weekend patient population, making it difficult to draw conclusions about the implications of this effect.

**Methods:**

A retrospective cohort study, examining de-identified, patient level data from 2015 to 2017 at US academic medical centers submitting data to the Vizient database, comparing demographic and clinical risk profiles, as well as mortality, cost and length of stay, between weekend and weekday patient populations. Between-group differences in mortality were assessed using the chi-square test for categorical measures and Wilcoxon rank-sum test for continuous measures. Logistic regression models were used to test the multivariate association of weekend admission and other patient-level factors with death, LOS, etc.

**Results:**

We analyzed 10,365,605 adult inpatient encounters. Within the weekend patient population, 30.6% of patients were categorized as having either a major or extreme risk of mortality on admission, as compared to 23.7% on weekdays (*p* < 0.001). We found a significantly increased unadjusted mortality rate associated with weekend admission (OR 1.46; 95% CI 1.45–1.47) which was substantially attenuated after adjusting for disease severity and other demographic covariates, though remained significant (OR 1.05; 95% CI 1.04–1.06). In the subgroup of non-elective admissions, the unadjusted OR for death was 1.14 (95% CI 1.13–1.15), and the adjusted OR was 1.04 (95% CI 1.03–1.05). Weekend admission was associated with a longer median LOS (4 vs 3 days in the weekday group; *p* < 0.01), but a lower median cost ($8224 vs $9999 dollars in the weekday group; *p* < 0.01).

**Conclusion:**

The patient population admitted on weekends is proportionally higher risk than the population admitted on weekdays, and the observed weekend mortality effect is largely attributable to this risk imbalance.

## Background

The weekend mortality effect- whereby patients admitted to the hospital on the weekend have a higher mortality rate than those admitted on weekdays- is a well-described, albeit controversial, topic in the literature. This association has been identified in several observational studies [1–7] and meta-analyses, [8, 9] ranging from broad population datasets to specialty and disease specific groups, though there are notable negative studies [10, 11]. The relative increased risk of death for weekend admission has varied across datasets, but has typically fallen between 10 and 20% [9, 12].

A particular point of contention has surrounded the root cause of the weekend mortality effect, with the prevailing lines of thought falling into either patient extrinsic (reduced weekend staffing, increased delays in time to necessary procedures, etc.) [9, 13, 14] or patient intrinsic (severity of illness, number of comorbidities, etc.) factors [15, 16]. Prior studies have been limited in their characterization of the weekend patient population, largely due to limitations of datasets [17]. For this reason the question remains unresolved.

The purpose of the present study was to examine the outcomes of weekend admissions in a large subset of US academic hospitals, while taking into account the unique features of the individual patients. We conducted a retrospective cohort study, investigating patient level characteristics and outcomes of weekend versus weekday admissions across a nationally representative sample of US academic medical centers.

## Methods

### Data source

We obtained patient level data from in-hospital admissions for academic hospitals submitting data to the Vizient clinical database between January 2015 and December 2017. (encompasses 106 academic hospitals across 39 states and Washington DC). Vizient maintains a clinical database which contains discharge and patient-level detail from member institutions. Member institutions submit data to the clinical database on a regular basis, primarily consisting of administrative billing records, supplemented by value-added data from Vizient such as clinical flags and 3 M grouper values for APR-DRG, severity of illness, and risk of mortality (see below).

All adult admissions (age 18 and older) were included for the primary outcome analysis. Subsequent analyses for both the primary outcome as well as secondary outcomes were conducted on the subgroup of non-elective admissions out of concern for unmeasured confounding. We excluded cases with an admission status documented as newborn or UHC service line code indicating either neonatology or normal newborns (935 cases, 76 cases and 25 cases, respectively), and all cases that did not have a risk of mortality or severity of illness specified (6984 cases). A small number of patient IDs had a mortality documented in more than one encounter (363 patients, < 0.01% of all encounters; 370 additional deaths); in these cases we included the last encounter and excluded the excess deaths.

Since we did not have a time of admission, patients were grouped according to day of admission, with weekend admission defined as patients admitted from midnight Friday to midnight Sunday [1, 2].

### Outcome measures

The primary outcome examined was in-hospital mortality, which is registered in Vizient based on coding of discharge status. Secondary outcomes included length of stay (LOS), total cost (direct and indirect) of admission, and hospital acquired conditions (HACs). Direct cost are those related to patient care, while indirect costs incorporate capital and building costs. Vizient provides an estimated direct cost by using a ratio of cost to charges combined with detailed patient charges submitted by member institutions. This ratio is based on the 2017 risk adjustment model, which incorporates the calculation of a hospital specific wage index to adjust for labor differences across the country. HACs are as defined by CMS, and include vascular catheter associated infection, catheter associated urinary tract infections, and stage 3 and 4 pressure ulcers, among others. The complete list can be found on the CMS website (https://www.cms.gov/medicare/medicare-fee-for-service-payment/hospitalacqcond/hospital-acquired_conditions.html).

### Independent variables

Analysis of outcomes took into account illness severity and risk of mortality at the time of admission. Illness severity and risk of death were defined as minor, moderate, major or extreme, based on the 3 M All Patients Refined Diagnosis Related Groups (APR-DRG) grouper. These 3 M levels are based on a proprietary algorithm. The variable inputs into the algorithm are patient age, sex, primary admission diagnosis (for medical patients) or procedure (for surgical patients), and secondary diagnoses/conditions. Based on these indices, 3 M has constructed, and subsequently validated, separate scales for severity of illness (defined as the extent of physiologic decompensation or organ system loss of function) and risk of mortality (likelihood of dying). Other patient characteristics included age, sex, number of comorbidities, race, ethnicity, primary payer (Medicare, Medicaid, commercial insurance, or other), and admission status (emergency, urgent, elective, trauma).

### Analysis

Between-group differences in mortality were assessed using the chi-square test for categorical measures and Wilcoxon rank-sum test for continuous measures. Logistic regression models were used to test the multivariate association of weekend admission and other patient-level factors with death, LOS, etc.. All statistical analyses were conducted in SAS version 9.4 (SAS Institute, Cary, North Carolina).

Because our study used de-identified patient data, available to all members of the Vizient database, our study was determined by the institutional review board to be not human subjects research, and therefore did not require further review.

## Results

Our analysis included 10,365,605 adult inpatient encounters for 6,777,415 patients at academic hospitals. Of these encounters, 8,408,723 (81.1%) occurred between Monday and Friday, while 1,956,882 (18.9%) occurred on the weekend (Saturday or Sunday).

### Demographics

The weekend patient population was slightly younger (median age 55.7 vs. 57.2 in the weekday group; *p* < 0.001) (Table 1). It had proportionally more self-identified black (24.7% vs. 21.6%; *p* < 0.001) and Hispanic (9.5% vs 8.7%; *p* < 0.001) patients, and fewer self-identified white patients (60.1% vs 64.1%; *p* < 0.001). Patients admitted on weekends were also more likely to have Medicaid (23.6% vs 20.6%; *p* < 0.001) and less likely to have private insurance (26.5% vs. 31.0%; *p* < 0.001).

**Table 1:** Patient Characteristics

Characteristics	Monday through Friday (***N*** = 8,408,723)	Weekend (Saturday/Sunday) (***N*** = 1,956,882)	***p***-value
	% or Median [IQR]	% or Median [IQR]	
**Age**			
*Median*	57.2 [38.9–69.5]	55.7 [36.1–69.9]	< 0.001
**Sex**			
Male	45.6%	46.0%	< 0.001
Female	54.4%	54.0%	< 0.001
Unknown	< 0.01%	0.01%	< 0.001
**Race**			
White	64.1%	60.1%	< 0.001
Black	21.6%	24.7%	< 0.001
Asian	2.8%	2.9%	< 0.001
Other	9.1%	9.7%	< 0.001
Unknown/Unavailable/Declined	2.5%	2.6%	< 0.001
**Ethnicity**			
Hispanic	8.7%	9.5%	< 0.001
Non-Hispanic	76.6%	76.6%	< 0.001
Unknown/Not Reported/Unavailable/Declined	14.7%	13.9%	< 0.001
**Primary Payer**			
Commercial	31.0%	26.5%	< 0.001
Medicaid	20.6%	23.6%	< 0.001
Medicare	40.4%	40.5%	< 0.001
Other	8.1%	9.4%	< 0.001
**Admission Risk of Mortality**			
Minor	48.8%	42.4%	< 0.001
Moderate	27.5%	27.0%	< 0.001
Major	18.0%	21.9%	< 0.001
Extreme	5.7%	8.7%	< 0.001
**Admission Severity of Illness**			
Minor	24.0%	19.0%	< 0.001
Moderate	38.7%	36.3%	< 0.001
Major	29.2%	33.5%	< 0.001
Extreme	8.2%	11.2%	< 0.001
**Admission Status**			
Emergency	49.0%	70.2%	< 0.001
Urgent	17.6%	17.8%	< 0.001
Elective	31.4%	8.3%	< 0.001
Trauma Center	1.6%	3.3%	< 0.001
Info not available	0.3%	0.4%	< 0.001
**Number of Comorbidities**			
*Median*	2 [1–4]	2 [1–4]	< 0.001
*Mean*	2.4	2.7	< 0.001

Patients admitted on weekends were also more likely to have markers of poor health status than those admitted during weekdays. The mean number of comorbidities was slightly higher (2.7 [+/− 2.1] vs 2.4 [+/− 2.0]; *p* < 0.001); a higher proportion of patients had a severity of illness categorized as major or extreme (44.7% vs 37.4%; *p* < 0.001) and a mortality risk categorized as major or extreme (30.6% vs 23.7%; *p* < 0.001). There were proportionally more urgent and emergent admissions (88.0% vs 66.6% in the weekday group; *p* < 0.001), and fewer elective admissions (8.3% vs 31.4% in the weekday group; *p* < 0.001).

### Primary outcome

There were a total of 250,953 deaths (2.4% of encounters; 3.7% of individuals) reported across all encounters. Of these, 187,822 (2.2% of encounters) occurred on weekdays and 63,131 (3.2% of encounters) on weekends (crude OR of 1.46; 95% CI 1.45–1.47) (Table 2). After multivariate adjustment for severity of illness, risk of mortality, number of comorbidities, and demographics this relationship was attenuated, though remained significant (OR 1.05; 95% CI 1.04–1.06).

**Table 2:** Primary Outcome

Primary Outcome	Monday through Friday (***N*** = 8,408,723)	Weekend (Saturday/Sunday) (***N*** = 1,956,882)	***p***-value	Crude OR* (95% CI)	Adjusted OR (95% CI)
	n (%)	n (%)			
**Death**	187,822 (2.2)	63,131 (3.2)	< 0.001	1.46 (1.45–1.47)	1.05 (1.04–1.06)

Specific patient-level factors that were significantly associated with death were risk of mortality (AOR 1.07, 95% CI 1.06–1.08), and admission status (AOR of 1.08, 95% CI 1.07–1.09).

### Subgroup analysis

In the subgroup of patients non-electively admitted, there were a total of 7,559,722 encounters for 4,942,479 patients. There were a total of 236,796 deaths (3.1% of encounters, 4.8% of individuals) reported across these encounters. Of these, 174,998 (3.0% of encounters) occurred on weekdays and 61,798 (3.4% of encounters) on weekends (crude OR of 1.14; 95% CI 1.13–1.15) (Table 3). After multivariate adjustment for severity of illness, risk of mortality, number of comorbidities, and demographics this relationship was attenuated, though remained significant (OR 1.04; 95% CI 1.03–1.05).

**Table 3:** Subgroup of Non-Elective Admissions

Outcome	Monday through Friday (*N* = 5,764,339)	Weekend (Saturday/Sunday) (*N* = 1,795,383)	***p***-value	Crude OR* (95% CI)	Adjusted OR (95% CI)
	n (%)	n (%)			
**Death**	174,998 (3.0)	61,798 (3.4)	< 0.001	1.14 (1.13–1.15)	1.04 (1.03–1.05)
**LOS (days)**	4 [2–7]	4 [2–6]	< 0.001		
**Total Cost (USD)**	8639 [4831 – 17,361]	8348 [4755 – 16,900]	< 0.001		
**HAC (%)**	0.3	0.3	0.02		

### Secondary outcomes

#### Length of stay

Median length of stay in the overall population was longer for patients admitted on weekends at 4 days (median IQR [2–7]) versus 3 days (median IQR [2–6]) for the weekday group (*p* < 0.001) (Table 4). Median length of stay in the group of patients admitted non-electively was similar for patients admitted on weekends (4 days, median IQR [2–7]) and weekdays (4 days, median IQR [2–6]) (*p* < 0.001) (Table 4). After multivariate adjustment, and excluding encounters that resulted in death, the length of stay was shorter for patients admitted on weekends (difference, − 2.87, 95% CI: − 3.06, − 2.69%, *p* < 0.001).

**Table 4:** Secondary Outcomes

Secondary Outcomes	Monday through Friday		Weekend (Saturday/Sunday)		***p***-value
	No. Observed	Median [IQR]	No. Observed	Median [IQR]	
**LOS (days)**	8,408,721	3 [2–7]	1,956,882	4 [2–6]	< 0.001
**Total Cost (USD)**	8,219,677	9999 [5459 – 19,247]	1,907,171	8224 [4732 – 16,696]	< 0.001
**Hospital Acquired Conditions**	8,408,723		1,956,882		
HAC criteria is not met		8,385,152 (99.72)		1,951,178 (99.71)	< 0.001
One or more HAC criteria met		23,571 (0.28)		5704 (0.29)	< 0.001

#### Cost

Median cost in the overall population was $8224 for the weekend group (median IQR [4732 – 16,696]) and $9999 for the weekday group (median IQR [5459 – 19,247]) (*p* < 0.001). Median cost in the group of patients admitted non-electively was $8348 for the weekend group (median IQR [$4755 – $16,900]) and $8639 for the weekday group (median IQR [$4831 – $17,361])(*p* < 0.001) (Table 4). After multivariate adjustment, total cost in the weekend admission group was slightly less than that in the weekday group (difference, − 0.97, 95% CI: − 1.19, − 0.76%, *p* < 0.001).

#### Hospital acquired conditions

Of the cases reporting on occurrence of HACs, 99.7% reported no HAC in both the weekend and weekday admission groups. There were a total of 22,423 HACs, accounting for 0.30% of all admissions. HAC rates were equivalent in the weekend (0.30%) and weekday (0.30%) admission groups (*p* = 0.02).

## Discussion

In this study of over 10 million hospital admissions in US academic medical centers in 39 states, patients admitted on weekends had a higher risk of death than those admitted on weekdays. While we found a significant, unadjusted increased risk of mortality associated with weekend admission (odds ratio of 1.46), after adjusting for patient risk profile and demographics, this was attenuated to a much more modest, though still significant, effect (odds ratio 1.05). Our findings were similar when looking at the subgroup of non-electively admitted patients (AOR 1.04). This result suggests that the observed weekend mortality effect in US academic medical centers is largely a product of the unique demographic and risk profile of the weekend patient population, a conclusion that many earlier studies did not reach, [2, 5] and only recently has gained traction [15, 16, 18]. We found a significant difference in the patient risk pool, with the weekend population consisting of proportionally higher risk patients; correspondingly, the largest confounding effect was seen when adjusting for patient admission risk of mortality.

We also found several other unique features of the weekend patient population, including a greater proportion of Medicaid insured patients and a greater proportion of patients that self-identified with historically underserved minority groups (in particular black and Hispanic). The magnitude of all of these demographic differences is relatively small, but may reflect challenges in access to non-hospital based care over the weekends in these groups.

Our study builds on other recent work that has begun to call into question the nature of the weekend mortality effect internationally [15] and in the US [18]. Ko et al. conducted a similar analysis on a cohort from the National Inpatient Sample and found a risk adjusted odds ratio of 1.029 (95% CI, 1.020–1.039; *P* < .0001), which, while significant, was a smaller effect size than seen in other settings, and in line with what we found. The notable discrepancies in our findings pertain to the unique characteristics of the weekend patient population described above- they did not find as pronounced a difference in proportional risk of mortality or severity of illness- which may be attributed to a few factors. For one, our analysis is focused on academic medical centers, whereas theirs included non-academic hospitals. They also chose to exclude elective admissions out of concern for unmeasured confounding. While we share this concern, we conducted our analysis both with and without elective admissions included for a couple of reasons. First, we maintained elective admissions in our primary analysis due to lack of standardization across systems in classifying elective and non-elective admissions. For those systems that define elective based on referral from an outpatient source, this misses many emergent cases [19]. We also maintained all admission sources to more accurately replicate earlier work that had taken a broader, more inclusive approach [19]. Our study population closely resembles that of Freemantle et al., one of the more widely cited papers, which looked, separately, at hospitals in the UK and a large subset of academic medical centers in the US. In the latter analysis they found an adjusted hazard ratio of 1.18 ([95% CI 1.11 to 1.26] *P* < .0001).

There are likely several reasons for the differences in our result, some of which are highlighted by Ko et al. These include the use of Wednesday as an index day against which to compare weekend days, when there is in fact some variation in outcomes across weekdays [10]. They also limit their analysis to in-hospital deaths within 30 days of admission. Most notably, however, they lack a validated metric by which to risk stratify their population. In our study, the 3 M severity of illness metrics allowed us to characterize a more acutely ill weekend patient population, and adjusting for this alone demonstrated a greater confounding effect than any other single covariate.

There are several potential explanations for the higher illness severity observed among weekend compared to weekday admissions. Meacock et al. [20] looked at the proportion of patients presenting to the ED that were admitted on weekends vs weekdays, finding a higher threshold of illness severity for admission on weekends. In other words, the admission triage process on weekends may simply select a sicker subset of patients. Another possibility is that patients generally avoid coming into the hospital on weekends unless they feel they must, creating a natural selection pressure for more acute patients; this, however, is not supported by Meacock’s findings. There are, of course, far fewer elective admissions on weekends (in our study population, 8.3% vs. 31.4% on weekends and weekdays respectively) with most outpatient practices closed and scheduled procedures not occurring, and at face value one would expect these patients to be less acutely ill; indeed, they have a much lower mortality rate than those admitted urgently or emergently. However, admission status alone does not account for the weekend effect, further suggesting a self-selection process among those presenting on weekends.

The difference in cost between the weekend and weekday groups, though statistically significant, is quite small in magnitude, and unlikely to be interpreted as meaningful. While the prior literature is conflicting on this question, [5, 18, 21, 22] our findings are in line with similarly designed studies that have taken a broader approach. Finally, the difference in LOS between the weekend and weekday groups is minimal (2.9% shorter in the weekend group) after multivariate adjustment. There has been no clear consensus on this question across the literature, though, in general the differences between weekend and weekday groups have been similarly small [5, 8, 10, 23]. This may reflect the reality that length of stay, as an outcome, is influenced not only by patient complexity and illness factors, but also by discharge processes that often involve complex logistics and incorporate essential ancillary staff (whose services are even more dramatically reduced over weekends). Fonarow, et al., found the longest LOS associated with Thursday and Friday admissions, for example; for admissions whose median LOS is between 3 and 4 days, those admitted late in the week may suffer the most from the limited discharge coordination available on weekends [10].

Limitations of our study include the fact that, like many other studies in this space, this is a retrospective cohort study, reliant on accurate, consistent coding across many EMRs and institutions. Because our primary exposure of interest is day of admission, there is no reason to believe that any flaws in coding or documentation would affect any particular cohort more than another. For this reason, we considered as broad a population as possible in our analysis, and then stratified by, rather than excluded, various designations. While a major strength of our study is the ability to account for patient intrinsic factors, using metrics for risk of mortality and severity of illness, our dataset is limited in its inability to assess extrinsic factors, such as delays in care and reduced weekend staffing. The demographic differences we found between weekend and weekday populations comes with the caveat that EMR reporting of race and ethnicity is fraught with error, and data from retrospective studies such as ours using these variables should be interpreted with caution. Lastly, despite adjustment for a wide array of sociodemographic covariates, there is always the potential for residual confounding.

## Conclusion

The weekend patient population admitted to the hospital has several unique characteristics, including a proportionally higher risk profile. The higher mortality rate seen in this group is significantly attenuated by adjusting for these factors. The demographic profile of weekend admissions to the hospital may reflect particular challenges in access to non-hospital based care on weekends in certain groups. Given that the weekend patient population is an inherently higher risk population, it is not clear that infrastructural changes related to expanded staffing will significantly impact the outcomes in this group.

## Data Availability

The datasets used and/or analyzed during the current study are available from the corresponding author on reasonable request.

## Abbreviations

AOR: Adjusted odds ratio
APR-DRG: All patients refined diagnosis related groups
CI: Confidence interval
EMR: Electronic medical record
HAC: Hospital acquired condition
LOS: Length of stay
OR: Odds ratio
